# RASI’S IN RECOMMENDED HIGHER TARGET DOSES MAY LOWER RISK OF DEATH AND KIDNEY FAILURE IN OCTOGENARIANS WITH HFREF

**DOI:** 10.1093/geroni/igae098.4365

**Published:** 2024-12-31

**Authors:** Ali Ahmed, Paul Heidenreich, Wilbert Aronow, Richard Allman, Charles Faselis, Mo-kyung Sin, Phillip Lam, Gregg Fonarow

**Affiliations:** Washington DC Veterans Medical Center, VA Medical Center, District of Columbia, United States; Stanford University, Stanford, California, United States; New York Medical College, Valhalla, New York, United States; George Washington University, Washington, District of Columbia, United States; Washington DC Veterans Medical Center, VA Medical Center, District of Columbia, United States; Seattle University, Seattle, Washington, United States; George Washington University, Washington, District of Columbia, United States; UCLA Health, Los Angeles, California, United States

## Abstract

Renin-angiotensin system inhibitors (RASIs) reduce the risk of death in patients with heart failure with reduced ejection fraction (HFrEF). National heart failure (HF) guidelines recommend that RASIs be used in higher target doses in patients with HFrEF. However, clinical trials of target vs. below-target dose RASIs did not include octogenarians, and target-dose group had worsening kidney function, but kidney failure (KF) was not examined. Thus, it is unknown whether RASIs in target doses may improve clinical outcomes without causing KF in octogenarians with HFrEF. Of the 311,611 Veterans with HF receiving RASIs, 32,964 were ≥80 years with ejection fraction ≤40% and without KF of whom 6655 (20.2%) received target-dose. Propensity scores for target-dose, calculated for each of 32,964 patients, were used to match 6642 (99.8% of 6655) target-dose patients with 6642 below-target-dose patients. Matched patients (N=13,284) were balanced on 66 baseline characteristics and had mean(SD) age 84.5(3.4) years, ejection fraction 31.3(8.2)%, and estimated glomerular filtration rate 58.5(18.2) mL/min/1.73m2. During 5 years of follow-up, 70.3% of the patients died, 34.1% were hospitalized for HF, and 1.7% developed KF. Target-dose RASI use was associated with a significant 5% (95% CI, 1–9%) lower risk of death (HR, 0.95; 95% CI, 0.91–0.99). There was a trend toward a lower risk of KF (HR, 0.80; 95% CI, 0.61–1.04), and consequently a lower risk of the composite endpoint of KF or death (HR, 0.94; 95% CI, 0.91–0.98). These new findings provide evidence for the use of RASIs in recommended higher target doses in octogenarians with HFrEF.

